# Quantifying antibody binding: techniques and therapeutic implications

**DOI:** 10.1080/19420862.2025.2459795

**Published:** 2025-02-16

**Authors:** James Lodge, Lewis Kajtar, Rachel Duxbury, David Hall, Glenn A. Burley, Joanna Cordy, James W.T. Yates, Zahra Rattray

**Affiliations:** a Large Molecule Discovery, GSK, Stevenage, UK; b Strathclyde Institute of Pharmacy and Biomedical Sciences, University of Strathclyde, Glasgow, UK; c Department of Pure and Applied Chemistry, University of Strathclyde, Glasgow, UK; d Preclinical sciences, DMPK, GSK, Stevenage, UK

**Keywords:** Antibody, affinity, avidity, target engagement, pharmacology, pharmacokinetics

## Abstract

The binding kinetics of an antibody for its target antigen represent key determinants of its biological function and success as a novel biotherapeutic. Defining these interactions and kinetics is critical for understanding the pharmacological and pharmacodynamic profiles of antibodies in therapeutic applications, with line of sight to clinical translation. In this review, we discuss the latest developments in approaches to measure and modulate antibody-antigen interactions, including antibody engineering, novel antibody formats, current, and emerging technologies for measuring antibody-antigen binding interactions, and emerging perspectives within the field. We also explore how emerging computational methods are set to become powerful tools for modeling antibody-binding interactions under physiologically relevant conditions. Finally, we consider the therapeutic implications of modulating target engagement in terms of pharmacodynamics and pharmacokinetics.

## Introduction

Monoclonal antibodies, inspired by their biological role in humoral immunity, represent a diverse and rapidly growing therapeutic category used to diagnose and treat a range of diseases by virtue of their tuneable biology. The flexibility of antibodies and derived biological therapeutics has made them an attractive modality for targeting previously intractable disease indications, with >250 entering clinical investigation in 2022,^1^ and half of the top 10 grossing therapeutics in 2024 predicted to be antibodies.^2^ The selection of the most appropriate antibody for a desired target represents a key determinant of clinical success and requires in-depth characterization of antibody biological attributes during early-stage drug development.

Integrated analysis of antibody binding, along with an understanding of pharmacokinetic/pharmacodynamic (PK/PD) properties during early antibody discovery efforts will improve the chances of successful clinical translation.^3^ These analytics include antibody disposition and exposure at the target site,^4^ target antigen expression levels,^5^ and antibody target engagement and occupancy rates.^6,7^ Here, we provide an industrial perspective on antibody affinity considerations, focusing on emerging developments in antibody engineering and models for target engagement, which are essential for achieving translational goals in antibody research and development efforts.

**Figure 1.**
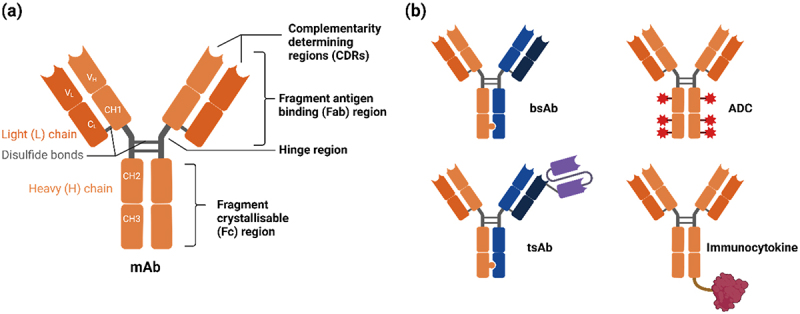
(a) Schematic depiction of the structure of IgG, the most frequently observed therapeutic antibody isotype. The antibody contains two heavy and two light chains, with disulphide bridges connecting the chains. The lower region, consisting of the CH2 and CH3 domains is frequently referred to as the Fc region. The upper region, containing the CH1 and VH regions of the heavy chain, and CL and VL regions of the light chain, are referred to as the Fab region. The VH and VL regions specifically include CDRs, which are key modulators of antibody target specificity and binding. These are separated by a flexible hinge region. (b) A selection of the best-represented antibody-derived therapeutic formats under investigation.

The function of an antibody is directly related to its structure, with different domains enabling interactions with antigens and other elements of the immune system.^8^ Native antibodies consist of two fragment antigen-binding (Fab) domains and a single fragment crystallizable (Fc) domain (Figure 1a). The Fab domains contain complementarity determining regions (CDRs), which mediate antibody target engagement through non-covalent interactions. Traditionally, the CDRs on both antibody arms are identical, but novel emerging formats (bispecifics) that combine different Fabs,^9^ multivalent Fabs, or single-chain variable fragments (scFvs) covalently attached to the antibody scaffold have been developed.^10,11^ Moreover, antibody conjugation to small-molecule drugs and peptides has given rise to antibody-drug conjugates (ADCs) and immunocytokines (Figure 1b).

The antibody Fc domain binds fragment crystallizable receptors (FcRs), *via* interactions between proteins and their associated glycans. IgG, the main therapeutic immunoglobulin isotype, binds specifically to Fc gamma receptors (FcγRs), though the affinity of this binding differs between IgG subclasses.^12^ Binding to FcγRs by higher-order antibody complexes induces receptor clustering and cross-phosphorylation, triggering downstream effects through immunoreceptor tyrosine-based activation and inhibitory motif (ITAM and ITIM) domains.^13^ Several sequence-based modifications and Fc domain glycan structures modulate affinity for specific receptors that either enhance or diminish specific effector functions.^14–16^ For example, obinutuzumab, a type II anti-CD20 monoclonal antibody, exhibits a higher affinity for CD20 than rituximab. Glycoengineering of the Fc region in obinutuzumab has led to a reduced core fucose content in its Fc region, enhancing its immune effector function compared to rituximab.

Recent observations have demonstrated that the binding properties of the Fc region can be modulated to some degree through contributions of the Fab region. Allosteric modulation involves conformational changes propagated to the Fc region resulting from antigen binding of the Fab region, altering antibody affinity for Fc receptors, which can enhance or deplete antibody-dependent cell-mediated cytotoxicity (ADCC) or phagocytosis (ADCP).^17,18^ Additional strategies include sequence engineering of the Fab or hinge region, to modulate flexibility and orientation of the Fc region, to enhance immune effector function. Additionally, introducing covalent bonds or optimizing non-covalent interactions between the Fab and Fc regions can stabilize specific conformations that enhance Fc receptor binding. Strategies to alter antibody Fc receptor engagement are reviewed elsewhere.^19,20^

The accurate determination of antibody-binding interactions is key to early discovery efforts, where structure–activity relationships are used as a proxy for progressing antibody candidates to later-stage development. Antibodies have been developed against a diverse range of targets, including soluble antigens, such as cytokines,^21^ growth factors,^22^ and hormones, and membrane-bound proteins, such as signaling molecules, and receptors.^23,24^ The nature of the target and indication of interest in a given antibody may dictate the affinity requirements and the target mechanism of action.

Consequences of antibody-binding events may be akin to small-molecule interactions in that they disrupt agonist:receptor complexes or directly antagonize signaling.^25^ Unlike small molecules, antibodies can interact with several therapeutic targets (Fc receptors), resulting in the recruitment and activation of immune effector cells, directly eliminating cells expressing the target antigen.^26^ Antibodies can also induce agonism by mimicking natural multi-valent ligands, driving receptor clustering and trans-activation.^27^ These diverse mechanisms of action and the emergence of novel antibody formats with increasing complexity render the examination of the relationship between antibody affinity and functional potency a complex task.

## Defining the relative contributions of antibody-binding interactions

Affinity describes the tightness of binding between two molecules. It is derived from the rates of association and dissociation, defining how quickly a complex assembles and disassembles.

$$\frac{\left[R][L\right]}{\left[\mathrm{RL}\right]}=\frac{k_{\mathrm{off}}}{k_{\mathrm{on}}}=K_{D}$$

In the context of antibodies, target affinity, which refers to the interactions between a CDR and its antigen epitope, is derived from association and dissociation rates that define the equilibrium rate of complex association and dissociation.^28^ Affinity can also be determined for other antibody domains, such as the Fc region. Due to high sequence homology in these constant domains, interaction strengths are generally consistent, with significant differences mainly observed between antibody isotypes or Fc receptor polymorphisms.^29^ The affinity and specificity of an antibody for its antigen are driven by non-covalent interactions, including hydrogen bonds, van der Waals forces, and electrostatic interactions mediated by amino acid residues in antibody CDRs.^30^

Determining and translating the affinity and steady-state behavior of an antibody targeting a solution-phase monomeric antigen, such as a chemokine or peptide neurotransmitter, is a simple process. In these cases, the two CDRs of a native antibody can be assumed to function as independent-binding sites. When fitting mathematical models to experimental data, the antibody concentration should be doubled to derive the concentration of antigen-binding sites. This approach also applies to bispecific antibodies targeting solution-phase ligands, where CDRs behave independently due to their distinct target antigens or epitopes.

Single affinity values (K_D_) are derived from association (*k*_*on*_) and dissociation rates (*k*_*off*_). Antibodies with similar affinities can have different kinetics. Therefore, it is worthwhile to understand the individual kinetic rate constants that feed into this K_D_ value when considering the observed therapeutic effect. This insight is useful in assessing how quickly an antibody binds to and dissociates from its target, impacting its overall efficacy and duration of action.

In drug discovery, determining antibody affinity is crucial for confirming target engagement, which underpins success in later stages of antibody development in terms of meeting clinical trial endpoints. However, target engagement must be verified for each antibody on a case-by-case basis, irrespective of previous in-class findings.^3^

### Avidity

By virtue of their bivalent format, antibodies concurrently engage two distinct antigens, a concept termed avidity. Avidity arises from co-localization of binding sites resulting in both antibody CDRs being bound to the same object, such as antigens on a cell membrane. This can be presented as an apparent increase in affinity, resulting from an enhanced rebinding potential, as a second Fab:antigen interaction can occur prior to dissociation of the primary complex.^31^ This necessitates the need for careful consideration and modeling when translating affinity estimates between *in vitro* and *in vivo* parameters.^32,33^

Avidity may occur for two reasons. First, both arms of an antibody are bound to spatially related sites: for example, epitopes on repeated domains of a protein, or to antigens tethered to a cell membrane. To fully dissociate, both arms must release their antigen, resulting in an effective dissociation half-life that is much longer than that of the antibody arms in isolation.^34,35^ Second, when bound to a cell surface-associated antigen, an antibody is spatially constrained. A potential consequence is that the second binding event is defined by the local availability of a second copy of antigen on the cell surface.^33,36^ Further, membrane association can result due to a higher effective concentration of antibody with reference to that in solution, as well as the second association rate being driven by the rate at which target antigens come into close proximity.^32^ This second set of assumptions has been validated against published data for bispecific antibodies.^37^ Thus, avidity can be viewed as a form of cooperativity, where the binding of one arm results in a change in the binding kinetics of the other arm.^31^ The avidity effect due to higher effective antibody concentrations on the cell membrane would therefore be dependent upon cell density and antigen expression level.

**Figure 2.**
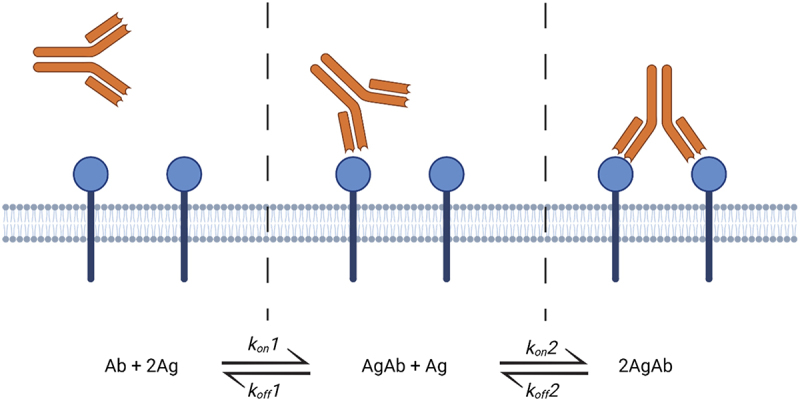
Antibodies are bivalent and can bind multiple targets through avidity. For monospecific antibodies, both arms have the same affinity, and the strength of the second binding event is enhanced by the constraint imposed by the primary binding event.

Avidity can be advantageous when the goal is to maximize target engagement with the intent of antagonizing target antigen biological function. However, when the aim is to direct the immune systems, such as with ADCC and ADCP, predominately bivalent interactions might be limiting. Experimental observations have shown that engineered antibodies that are either monovalent or have lower intrinsic affinity can be more potent or exhibit an apparently increased E_max_ due to increased antibodies bound to the cell membrane (Figure 2).^38,39^

The concept of avidity, along with further considerations for antibody design and effector function, has been comprehensively reviewed elsewhere.^31^

## Modulating antibody-antigen interactions

Several approaches have been explored in recent years to modulate antibody target engagement. Enhanced antibody:target engagement has traditionally been achieved through improving antibody affinity or by altering valency and introducing additional functional domains that yield antibodies with sub-nanomolar affinities.^11^ As the limit of affinity optimization is reached, alternative approaches are required to further enhance target engagement, which we discuss in this review.

### Modulating antibody target affinity by sequence engineering

**Figure 3.**
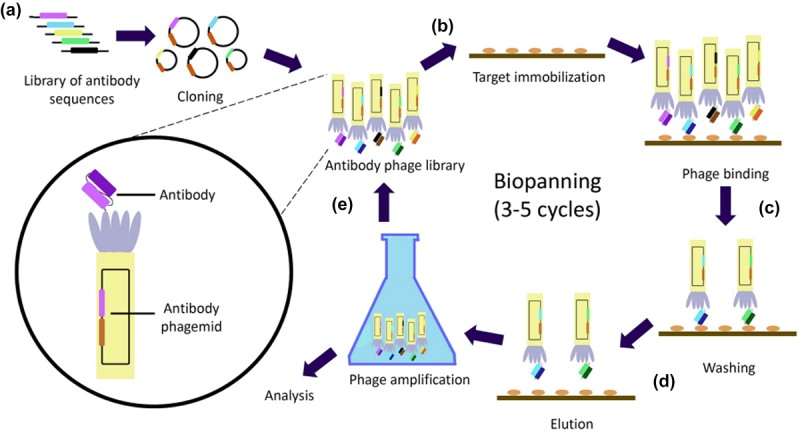
Schematic overview of phage display. (a). Libraries of bacteriophage expressing surface antibodies are generated from the transfection of bacteria with phagemids containing antibody coding sequences. (b). A target of interest is immobilised, and the naïve phage library applied. (c). Phages encoding non-specific antibodies are washed off while those encoding target specific antibodies remain bound. (d). Bound phages are eluted, amplified, and analysed. Relative binding affinity may be analysed using flow cytometry and antibody encoding nucleic acids sequenced. (e). 3-5 rounds of biopanning are commonly performed in which high affinity antibodies re-enter the phage display cycle until sufficiently enriched.^46^

The earliest point at which antibody affinity may be modulated is during discovery campaigns. Traditionally, modulation of antibody affinity has been achieved *via in vitro* phage, yeast, and mammalian display-based models for antibody discovery (Figure 3).^40–48^ Using these approaches, large libraries of naïve antibodies are screened for their target antigen-binding affinities.^40–48^ Affinity modulation can be achieved *via* artificial affinity maturation, where mutations are introduced to generate a mutant library with manipulated affinities for target binding.^45,47,48^ Polymerase chain reactions are commonly used to introduce site-specific or random mutations using degenerate oligonucleotides or error-prone polymerases, respectively.^45,47,48^ This process is repeated until the desired affinity for target binding is met. Furthermore, high-affinity antibodies can be selected by introducing harsher washing conditions and light-chain shuffling.^47,49^ *In vitro* approaches overcome the ethical concerns associated with animal use and enable greater governance of binding affinity. The modulation of binding affinity at this stage using *in vivo* approaches, however, remains prevalent.^40,41,50–61^

Using natural affinity maturation, *in vivo* models do not permit as great a degree of control over binding affinity as their *in vitro* counterparts, but they can generate high-quality antibodies with enhanced therapeutic characteristics.^62^ This has led to the establishment of *in vivo* techniques to modulate the discovery of high-affinity antibodies encoded by memory B cells and plasma cells. Immunization regimes may initially vary in numerous aspects to give the greatest prospect of stimulating high-affinity antibody generation.^40,41,50–61^ These include the format of immunization agent, adjuvant, pre-treatment, immunization regime, route of administration, and target organism.^40,41,50–61^ The function-first screening of *in vivo*-derived cells encoding antibodies enables the selection of high-affinity immunoglobulins including class-switched antibodies, and those exhibiting a high degree of antigen binding.^52,59,60^ Further innovations include the use of transgenic organisms expressing humanized antibodies capable of binding targets with high affinity and with greater prospects of translation to the clinic.^41,51,57,61^ Both *in vivo* and *in vitro* approaches for the discovery and selection of high-affinity antibodies have been further enhanced by next-generation sequencing (NGS).^40,41,43–45,63,64^

Established in 2000, the high throughput sequencing of nucleic acids *via* NGS has augmented the discovery of high-affinity antibodies by enabling the identification of nucleic acids that are likely to encode high-affinity antibodies based on their sequence similarity and features.^40,41,43–45,64^ NGS allows the screening of nucleic acid encoding antibodies at a previously unprecedented scale.^40,41,43–45,63,64^ Further, recent advances in artificial intelligence and machine-based learning (AI/ML) have augmented the ability of NGS to interrogate greater quantities of sequences for high-affinity binding with greater accuracy and have demonstrated capability for antibody, gene clustering, *de-novo* design, optimization, and modulation of binding affinity.^64–70^

### *In silico* affinity optimisation

The combinatorial use of NGS and AI/ML for antibody discovery has enabled high throughput screening and characterization of immunoglobulin encoding nucleic acids.^65–68,71^ Provided that a wealth of antibody sequences and binding data against an antigen pre-exists, AI/ML algorithms can be trained to mine large libraries of nucleic acid sequences and predict those likely to encode antibodies with the desired binding profiles.^68,71,72^ Further training may enable the optimization of antibody affinity through the prediction of mutations likely to modulate target binding.^72,73^ AI/ML has also advanced the *de-novo* discovery and design of immunoglobulins utilizing only target information to construct target-specific antibodies of desired affinities.^66–68,71,74^ *De novo* models for antibody discovery avoid the time, resourcing, and ethical concerns associated with classical *in vitro* and *in vivo* models; however, more complex AI/ML algorithms and training are required.^63–65,68,71^ AI/ML and NGS, therefore, have enabled the refinement of classical approaches for antibody discovery and modulation of binding affinity, as well as the establishment of revolutionary new approaches.

With the advent of *in silico* protein models and large pre-existing sequence and affinity data sets from historic antibody discovery campaigns, substantial effort has been invested in bringing these together to predict antibody affinity from sequences. A summary of AI/ML models is presented in Table 1.

**Table 1.** A summary of computational models used for antibody discovery.

Model Type	Description	Advantages	Disadvantages	Examples
***Deep learning models***^75^	Antibody-specific language models trained on extensive antibody sequence datasets, enabling high accuracy predictions of antibody-binding affinity. These models leverage the power of large-scale data and advanced machine learning techniques to improve antibody design. Capable of handling unpaired and paired variable region sequences as input, with applications in *de novo* antibody design, antibody optimization, antibody affinity prediction, epitope, and paratope mapping as well as enhancing developability of antibodies. IgT5‘s architecture allows it to handle a broader range of tasks with more versatility in a unified text-based approach, whereas IgBert focuses more on context within sequences.	Increased efficiency in generating high-quality antibodies. Enhanced specificity for the target antigen. Can generate novel designs without being limited to structures. Cost-effective. Lead candidate diversity.	Require large training datasets. Complexity and interpretability. Computationally intensive. Risk of overfitting. Require validation datasets. Requires expertise.	Bidirectional Encoder Representations from Transformers (IgBert) Text-to-Text Transfer Transformer (IgT5)
***Attention networks***^76^	Uses progressive encoding to integrate structural, residue-level, and sequential information, capturing both topological and contextual features of antibody-antigen interactions, resulting in accurate predictions of binding affinity changes caused by mutations.	Improved accuracy. Can indicate specific antibody sites benefitting. Can be scalable for large datasets. Can be trained more efficiently than traditional models.	More complexity to the model architecture. Computationally intensive. Risk of overfitting. Requires expert knowledge for data handling. Integration with other models can be challenging.	Antibody-Antigen Complex Attention Network (AbCAN)
***Bioinspired models***^77^	Trained on vast datasets of antibody sequences, capturing unique and conserved properties specific to antibodies. BALM can predict full atomic antibody structures from individual sequences, outperforming established methods like AlphaFold2, IgFold, ESMFold, and OmegaFold. Primary applications of BALM are sequence-structure-function predictions, which can be directly applied to the rational design and optimization of antibody engineering workflows.	Draw inspiration from nature. Enhanced specificity and affinity. Increased diversity of lead candidates. Reduce the need for library screening and immunization.	Complex to develop and implement. Computational resource. Require large and diverse datasets for training. Risk of overfitting. Challenging to interpret with complex structures. Challenging to integrate with other machine learning workflows,	Bio-inspired antibody language model (BALM)
***Generative models***^67,78–80^	AbDesign can be used to generate initial novel antibody sequences and structures, followed by AbDock to screen and optimize these designs for high binding affinity. The implementation of these generative tools in tandem can significantly accelerate antibody optimization efforts.	Can explore a diverse space of sequences. Enhanced diversity and rapid generation of a large number of sequences. Can be combined with other *in silico* approaches to refine antibody design. Can be adapted to new targets.	Not all candidates will be of high quality. Require large training datasets. Training can be computationally intensive. Risk of overfitting.	AbDesign AbDock RFdiffusion

The future success of AI/ML approaches for antibody engineering and optimisation is reliant on the availability of large antibody sequence datasets, along with their biophysical parameters, including antigen engagement. There are several notable repositories of paired and unpaired antibody sequences, such as the Observed Antibody Space (OAS) database and Patent and Literature Antibody database (PLAbDab).^81,82^ Associating these sequences with other molecular characteristics to train a model for affinity optimisation requires data that is only feasibly obtainable from employing high throughput screening techniques, as demonstrated in the case of anti-SARS-COV-2 antibodies.^83^

At the time of this review, no commercial antibody products in the clinic are reported to have been designed using the AI/ML-based approaches described above. The future utility of AI/ML approaches in therapeutic antibody design hinges upon the successful curation of datasets and data sharing within the pre-competitive space. Moreover, there are a small number of data repositories available for antibody docking and affinity predictions (e.g., antibody docking and affinity benchmark).^84^ Limitations associated with these repositories include limited diversity, generalisability of datasets to other antibody structures, and their sustainable legacy in terms of updating with novel datasets.

## Modulating target engagement *via* antibody structure engineering

Beyond target affinity, antibody target engagement can be enhanced by engineering the structure beyond the native monospecific format. Various approaches have been explored, including the pairing of two distinct CDRs within a single molecule to create bispecific antibodies. Additionally, the incorporation of functional domains has led to the development of more complex formats such as trispecific antibodies and immunocytokines. The diversity of formats currently under investigation has been explored in other reviews.^85,86^

Bispecific antibodies (bsAbs) are a class of therapeutic antibodies capable of targeting two or more different antigens or multiple epitopes on the same antigen. Unlike monospecific antibodies, bsAbs consist of two distinct Fab domains (Figure 1a). Not all bsAbs adhere to the standard IgG configuration, and various bsAb format configurations are under investigation.^87–89^ Multispecific antibodies aim to improve existing biopharmaceuticals by enhancing selectivity or efficacy. Due to their dual specificity, bsAbs may achieve obligate mechanisms that conventional antibodies cannot, such as cell redirection or pathway modulation.^87^ BsAbs can target two distinct cells (trans binding) or bind two targets present on the same cell (cis binding).^87^

**Figure 4.**
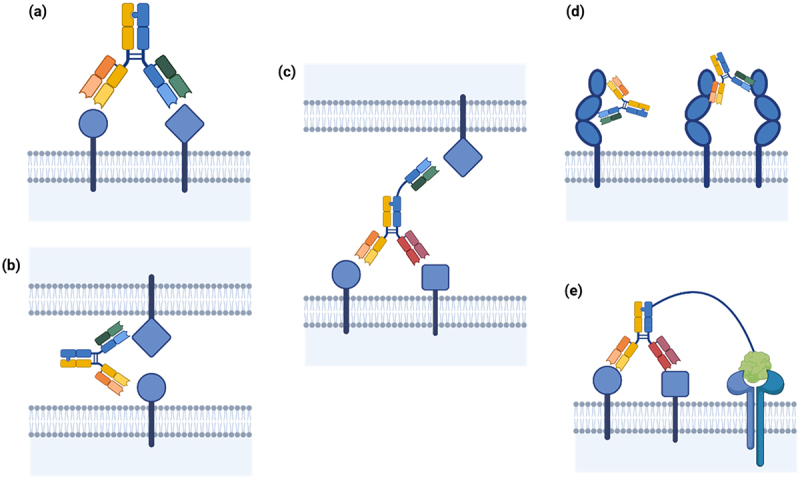
(a) Schematic of a cis binding mode, in which both Fab domains engage with two cell surface receptors, or two domains on a single protein. (b) Schematic of a trans binding mode, in which both Fab domains engage with antigens on distinct cells, or two distinct antigens in solution. (c) Schematic of a trispecific antibody, engaging in cis on the target cell, and engaging in trans on the effector cell using a covalently bound Fab. (d) A biparatopic antibody engaging two epitopes on the same target, both within the same antigen, and bridging between two antigens. (e) An immunocytokine binding in cis on the target cell, used to selectively target cytokine engagement also in cis.

*Cis binding* (Figure 4a) enhances antibody selectivity for cells that co-express two antigens, minimizing on-target/off-tumor toxicity. By reducing the affinity of each CDR, dissociation occurs more rapidly, decreasing binding to cells expressing only a single antigen. Selectivity relies on avidity to stabilize the binding interaction, which can be achieved by incorporating another low-affinity CDR event for a second, co-expressed target. Careful tuning of each arm’s affinity is crucial, as a high-affinity interaction can result in a stable complex with only one antigen bound,^90^ negating the benefit of avidity.^91^ The advantages of fine-tuning affinities in this manner have been demonstrated *in vitro* ^37,92^ and replicated *in vivo*.^93,94^

*Trans binding* (Figure 4b) is common with bsAbs used as immune cell engagers, where one arm targets the tumor-associated antigen (TAA) and the other engages an activating receptor on an immune cell, such as CD3 for T-cell engagers, or CD16A for natural killer (NK) cell engagers. Reducing the affinity for CD3 in T-cell engagers enhances selectivity and reduces cytokine release syndrome.^92^

Recent engineering strategies have explored trispecifics (Figure 4c), which combine the benefits of cis binding for selectivity, and trans binding to engage an immune receptor. They can also enhance cis selectivity by increasing avidity through triple-expressing cells.

*Biparatopic antibodies* (Figure 4d), a specific class of bsAbs, bind two distinct epitopes on the same antigen simultaneously. These antibodies improve receptor internalization, increase lysosomal trafficking, and promote degradation,^95^ which may be advantageous for ADCs.^96,97^ By exploiting two non-overlapping epitopes, it is possible to achieve greater antibody engagement with a target cell, increasing the density of Fc domains presented and therefore enhancing the Fc-mediated effector function.^98^

*Immunocytokines* (Figure 4e) resemble ADCs in design but carry a cytokine payload to specifically activate the cognate receptor in cis. Given the safety concerns historically associated with cytokine administration, immunocytokine design must ensure high antibody-binding specificity to the target, and selectivity for disease-associated cells along with attenuated cytokine affinity to minimize effects in the absence of antibody binding.^99^

## The importance of epitopes in antibody binding kinetics

A crucial consideration in correlating antibody affinity to effector function is the epitope(s) to which the antibody binds. Some epitopes can be considered “productive”, in that antibody engagement has a functional consequence, such as ligand neutralization, immune cell engagement, or ADC internalization. In contrast, the engagement of “non-productive” epitopes may result in no therapeutic efficacy observed, which may occur due to unfavorable antibody presentation for immune engagement, or if the epitope is not involved in activation of the downstream signaling cascade. It follows, therefore, that high affinity may be a red herring if strong binding does not lead to a favorable therapeutic output. Therefore, targeting and inhibiting critical residues, such as those in a receptor or ligand’s binding domain or in an optimal orientation for immune cell engagements, is more effective than targeting distal sites.

Epitopes can be profiled within a panel of antibodies using high-throughput competition-based assays.^100^ Such assays, based on flow cytometry or surface plasmon resonance (SPR), measure the ability of antibodies to outcompete each other for binding, thereby inferring that competition arises from steric hindrance due to a common epitope. While these approaches are suitable for screening and ensuring epitope diversity in a discovery campaign, higher resolution studies such as hydrogen deuterium exchange (HDX) mass spectrometry can provide further insights into the specific residues involved in an interaction. There is a growing body of evidence supporting the use of antibody combinations that bind non-competing epitopes on the same target to achieve greater target engagement and therefore improved therapeutic potency.^101–103^

Epitopes can also modulate the likelihood of avid target engagement. Specifically, certain epitopes may enhance or hinder the presentation of antibody CDRs to a second copy of antigen. This phenomenon is exemplified by type I and II anti-CD20 antibodies, which differ in their ability to cluster antigen.^104,105^

## Measuring antibody-antigen interactions

The selection of biophysical techniques for measuring antibody affinity is guided by the target, and complexity of the binding partner interactions to avoid misinterpretation of target engagement. In this section, we discuss gold-standard techniques and emerging advances in antibody affinity measurements, categorizing them according to those using immobilized ligands and on-cell-based assays. A summary of assay technologies, and the advantages and disadvantages of each, is presented in Table 2.

**Table 2.** An overview of current and emerging techniques used to measure antibody affinity.

Method	Format	Advantages	Disadvantages	Affinity limit	Ref
Surface plasmon resonance (SPR) and surface-based fluorescence approaches	Ligand-based	High throughput Label-free Kinetic	Limited to low pM affinity Immobilized binding partner Mass transport	mM-pM	Hearty, et al.^106^ Matharu, et al.^107^
Biolayer Interferometry (BLI)	Ligand-based	High throughput Label-free Kinetic measurement	Less sensitive than SPR for low-affinity measurements Requires high sample volumes May struggle with accurately measuring very tight binders or interactions with very fast on-rates and off-rates due to diffusion limitations Requires cross-validation with another technique	mM-pM	Santos-López, et al.^108^
Flow cytometry	On-cell	High throughput Suitable for membrane targets	No kinetics May not be at equilibrium Live cell dynamics may complicate measurement	mM-nM	Hunter and Cochran^109^
Enzyme-linked immunosorbent assay (ELISA)	Ligand-based	High throughput	Immobilised	mM-nM	Bobrovnik^110^
Kinetic exclusion assays (KinExA)	On-cell and ligand based	Label-free High sensitivity (fM) Suitable for soluble and membrane targets Low sample consumption	Low throughput Live cell dynamics may complicate measurement	nM-fM	Erasmus, et al.^111^ Darling and Brault^112^
Single cell interaction cytometry (scIC)	On-cell	Kinetic measurement Suitable for membrane targets Single-cell resolution Native cell environment High sensitivity	Complex setup Limited throughput Novel Similar LOD to SPR Requires labelling	mM-pM	Harwardt, et al.^113^
LigandTracer	Ligand-based	Kinetic measurement Suitable for membrane targets	Low throughput Limited to surface-bound cells Complex sample preparation Potential for non-specific binding	pM-µM	Spiegelberg, et al.^114^
SwitchSense	Ligand-based	Kinetic measurement Label-free Multiplexing (two binding events) possible	Immobilization required Complex sample preparation Limited to DNA compatible molecules Potential for non-specific binding	pM-µM	Nowicka, et al.^115^
Functional assays	On-cell	Affinity studied in the context of functional response	Low throughput Complex setup Sample consumption dependent on affinity	fM	Issafras, et al.^116^
Mass photometry	Ligand-based	Other information about engagement can be gained, i.e., stoichiometry	Low throughput Complex setup Novel	nM-µM	Wu and Piszczek^117^ Kofinova, et al.^118^
Microscale Thermophoresis (MST)	Ligand-based	Low sample volume Wide dynamic range No immobilization required Rapid analysis High sensitivity Versatile in terms of buffer conditions	Requires fluorescent labelling No kinetic data No concentration analysis	pM-mM	Luo and Chen^119^

In the next section, we discuss these biophysical assays that rely on antibody or target antigen immobilization to mitigate for avidity effects. Each assay presents unique characteristics and conditions relating to their throughput, assumptions on binding kinetic models, dynamic ranges for K_D_ measurement, surface-based artefacts relating to loss of antigen three-dimensional native structure, and different immobilization chemistries.

### Surface plasmon resonance

**Figure 5.**
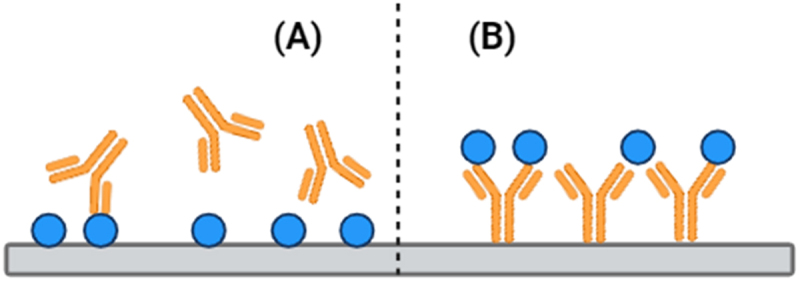
Schematic of an SPR chip surface in two assay design formats. In assay A, the binding partner is covalently immobilised onto the chip surface, with the antibody introduced in solution. Assay B shows the experiment in an alternative format in which the antibody is immobilised onto the chip surface, with the binding partner in solution.

SPR is widely used to measure the binding kinetics of antibodies to immobilized antigens, complement, or Fcγreceptors in real-time. This high throughput, label-free technique provides detailed insights into antibody-antigen binding kinetics, including association and dissociation rates.^120,121^ These assays can be configured in one of two ways, as depicted in Figure 5, by immobilizing either binding partner. Binding and dissociation events alter the refractive index of the gold chip where the analyte is immobilized, resulting in changes in light scattering intensity that can be measured in real-time.

However, SPR may lack physiological relevance when correlating *in vitro* and *in vivo* findings. Immobilized cell surface antigens may not accurately replicate their three-dimensional structure within cell membranes, and native antigen interactions in solution are constrained by immobilization, limiting the observed interaction freedom. Additionally, SPR experiments are typically designed to minimize avidity effects, enabling the 1:1 binding model, although some efforts have aimed to mimic target density on membranes.^36,122^ Furthermore, the buffer conditions used in SPR may not accurately reflect the physiological-binding environment in terms of pH, temperature, or composition.

Very slow dissociation rates can be challenging to measure by SPR due to limitations associated with prolonged washout protocols, which restrict affinity measurements to the low-picomolar range.^111^ This limitation can be mitigated by studying a range of antibody concentrations to ensure accurate-binding kinetics, but these limitations highlight the need for cross-validation of SPR experimental readouts with alternative, physiologically relevant systems.

### Flow cytometry

Cytometric on-cell binding assays are used for antibody titration against a fixed number of cells expressing target antigen. These assays use a fluorescently labeled ligand or labeled secondary antibody to detect binding, allowing determination of the concentration at which half-maximal binding is achieved (EC_50_) and maximum binding, which can provide insights into antibody-binding mechanisms and are generally analyzed based on a 1:1 equilibrium model.^109^ In contrast to immobilized ligand-based assays, the transmembrane antigen retains its three-dimensional structure enabling avid binding, and often results in biphasic-binding profiles. Consequently, it is important to avoid conflating observed EC50 values with affinity.

A limitation associated with cytometric endpoint assays is the assumption of full equilibration of binding, or that receptor concentration is larger than the K_D_. This can be addressed by cross-validation against kinetic cytometric assays (e.g., LigandTracer).^114^

The reported EC_50_ in cytometric assays generally correlates with affinity, with maximum fluorescence indicating target cell opsonization. However, the avidity of bivalent antibodies must be considered, and may manifest in such assays as an enhanced EC50, but reduced maximum fluorescence as fewer mAbs is required to engage all antigens.

A higher concentration of antibodies is required to achieve the maximum signal resulting in higher half-maximal concentrations in cells expressing high antigen levels. Cytometry-based assays are used to rank large antibody panels from discovery campaigns; therefore, it is important to select cell lines representative of antigen expression profiles observed in the target disease population.

There are several considerations when designing a cell binding method, including ligand depletion and equilibrium.^109^ Other limitations of cytometry-based assays include antigen:antibody complex internalization, resulting in observed antibody binding being reduced and reduced secondary antibody binding. Mitigation strategies include cell fixation or low-temperature incubation, though these may reduce the physiological relevance of any observations.

### Kinetic exclusion assay

Kinetic exclusion assay (KinExA) is a solution-based assay for determining binding partner concentrations and equilibrium dissociation constants of immune complexes. In antibody affinity measurements, the target antigen – whether membrane-associated or free in solution – is titrated into a constant concentration of antibody-binding sites, allowed to equilibrate, and subsequently exposed to antigen-coated beads under flow. The bead-captured antibody is detected with a fluorescently conjugated secondary antibody, enabling quantification of free antibodies at equilibrium and determination of antibody affinity.^112^ As these assays are typically allowed to reach equilibrium, they are considered to be more appropriate for measuring high-affinity, sub-nanomolar interactions

Although KinExa is lower throughput and shares limitations with fixed cell assays, it can be used in live cells. However, interference from media components such as endogenous IgG in bovine serum presents challenges in data interpretation due to the introduction of background signals and misleading quantification of IgG. There have been some efforts to correlate observations made using KinExA and SPR approaches, since they are considered to best represent solution-phase rate constants.^111^

### Single cell interaction cytometry

Single-cell interaction cytometry (scIC), also referred to as real-time interaction cytometry (RT-IC), is an emerging technique for the biorelevant analysis of antibody-antigen affinity and avidity, with the target antigen being in its native cellular environment.^113^ scIC uses live cells expressing the target antigen immobilized in a cage, with the fluorescently labeled test antibody passed under flow over the cell. The impact of enhancing the binding kinetics of affinity matured bispecific antibody mutants targeting EGFR and PD-L1 was explored using A431 and A549 cell lines. Enhanced binding affinity of several variants is correlated with slower dissociation rates and longer retention times on the cell surface.^113^

### Pharmacological functional assays for affinity determination

The role of pharmacological functional assays for affinity determination should not be overlooked where applicable. Antibodies can act as competitive antagonists of receptor:ligand complex formation, by binding to either the receptor or the ligand. This allows the determination of the antibody–ligand interaction affinity from the concentration-dependence of the observed response. In simple cases, a Cheng-Prusoff correction can be applied to the antibody inhibition curve to determine its affinity.^123^ However, caution is required with this approach, as it assumes competition and that the tight-binding limit of the system has not been reached. For more rigorous affinity determination, Schild analysis is superior since it can be used to identify issues with tight-binding and test whether the assumption of competitive behavior is reasonable. Such experiments have been described for the anti-IL-1B antibody gevokizumab,^116^ and for an anti-GLP-1R antibody,^124^ providing insights into the competitive nature of binding and the apparent affinity with which they outcompete their ligands’ native binding partner.^125^

## Therapeutic implications for function

**Figure 6.**
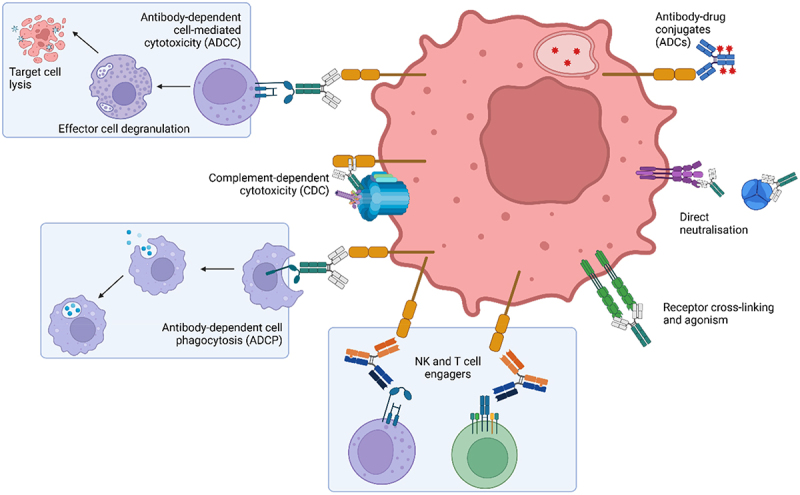
A summary of antibody mechanisms of action. Antibodies can elicit a range of effects, both directly for example by direct blockade of ligand binding to receptor or by inducing receptor clustering with subsequent activation of downstream signalling events, or indirectly by recruiting effector cells to the target cell as illustrated here by the recruitment of NK cells or phagocytes such as macrophages or neutrophils to induce target cell killing by ADCC or ADCP, respectively. Further cytotoxic mechanisms can be triggered by recruitment of complement to the cell surface or by engagement with alternative effector cells such as T cells. Antibodies can also be used to deliver a ‘payload’, commonly a toxin to target and kill tumour cells, by forming an ADC.

The affinity of an antibody for its target is generally considered to be closely correlated with its ability to induce a pharmacological effect. However, this correlation is not exclusively linear and is highly dependent on the specific pharmacological effect in question and whether it arises due to target occupancy or antibody density on the surface of the target cell (Figure 6).

### Tight binding and its implications for antibody function

High-affinity antibodies will often exhibit ‘tight binding’ to their target antigens, which is characterized by depletion of the free antibody from the solution phase when the binding partner concentration is in excess. Usually, it is assumed that when binding sites from the antibodies are present at the K_D_ concentration, 50% of binding sites on the antigen will be bound to the antibody (native antibodies are, of course, bivalent and this must be accounted for when calculating the relevant concentrations, as should antigen valency). However, this is only the case when K_D_ is greater than the antigen concentration, causing the antibody to be present in excess at the K_D_ concentration and hence avoiding its depletion. If K_D_ is lower than the antigen concentration in an assay, 50% occupancy can only be achieved when antibody-binding sites are present at, or above, half of the concentration of the antigen-binding sites rather than at K_D_. This situation is often referred to as “tight binding”, because in this scenario the majority of antibody is bound to antigen as 50% occupancy is approached, and half of the concentration of binding sites on the antigen is referred to as the ‘tight-binding limit’ of the system, as it is the lowest IC_50_ that can be measured

As the affinity of an antibody, or indeed any other ligand, approaches the tight-binding limit of an assay, its concentration–response curve will steepen and an inflection in the dose–response curve may be observed. The standard relationship between IC_50_ and K_D_ no longer applies under these conditions. Thus, the Hill-Langmuir and Cheng-Prusoff equations cannot be used to determine the affinity of an antibody under tight-binding conditions since these assume that the free concentration of the antibody is equal to the total concentration added to the system. The analysis should therefore be performed using a model which accounts for ligand depletion; for example, see Hulme & Trevethick.^125^

Antibody function typically follows target engagement and can be measured in many ways depending on the expected mechanism of action. In some cases, it may be expected that target engagement and antibody function share a linear relationship, with antibodies binding to their targets with greater affinity able to elicit effects at lower concentrations. The relationship between affinity and function may not always be clear, however, emphasizing the importance of screening for antibody function *in vitro* as part of a discovery screening campaign.

### Antagonistic antibodies

Antagonistic antibodies disrupt receptor signaling, either by direct receptor binding or sequestration of activating ligands. Their function may be assessed by observing downstream effects of target receptor activation. This can range from measuring signaling events such as receptor phosphorylation shortly after ligand binding, to downstream effects on cell proliferation or phenotype. Similarly, the neutralization of cytotoxic compounds such as bacterial toxins can be measured by comparing cell death when that compound is exposed to the titration of a specific antibody. Higher affinity antibodies will be able to engage their target and thus elicit their effects at a lower concentration, generally resulting in a linear relationship between target affinity and potency.

This is often true for antibodies that simply act to antagonize signaling pathways by disrupting ligand binding.^126^ There is likely some epitope-dependence here, as antibodies that bind to, for example, a receptor-binding domain will be more effective than those that bind to a more distal epitope, as observed in a panel of anti-SARS-CoV-2 antibodies.^127^

### Immune cell engagers

Immune cells engaging in antibodies cover a broad range of mechanisms of action, all of which induce target cell cytotoxicity, e.g., *via* ADCC, ADCP, or CDC. In addition to these traditional Fc-mediated effector functions, T cell and NK cell engaging antibodies have emerged more recently, recruiting immune cells by Fab-mediated targeting of specific effector cell immune receptors.^128^

Antibody effector function, whether mediated by FcγRs or other immune receptors, is often evaluated using recombinant cell systems, in which these immune receptors are coupled to a reporter gene (e.g., luciferase). This permits high-throughput screening and low inter-assay variability. Observations in these assays can then be validated in translationally relevant cytotoxicity assays employing human-derived immune cells or serum. Target cell death has been measured in a variety of ways – including pre-loading cells with fluorescent dye or radioligand (51-Chromium) or by measuring cell viability.

The relationship between affinity and function is not so clearly defined in these complex mechanisms of action, where antibody target engagement is required concurrently with, complement or FcγR binding and/or immune cell engagement. For such mechanisms, it may be useful to consider the Fc or other immune cell recruiting regions of the antibody as an agonist.

For Fc-dependent cytotoxic mechanisms, high-affinity antibodies may engage their target in an avid-binding mode, occupying two antigens with only a single Fc domain in return. In contrast, lower affinity antibodies may be more likely to bind monovalently, leading to greater cell opsonization, Fc presentation, and therefore immune cell and complement recruitment.^38,39^ Despite Fc structures being largely identical between antibodies of a given isotype, there may be some degree of Fab-mediated influence on Fc:FcγR interactions, both through the presence of specific residues which may interact with the Fc or FcγR, and their effect on the tertiary structure of the antibody resulting in an altered presentation of the Fc to FcγR.^18,129^

The functional activity of bispecific T cell and NK cell engagers is modulated by the affinity of both the TAA and T/NK cell-targeting domains.^130,131^ While the affinity for the TAA must be sufficiently high to engage the target, the affinity for CD3 must be carefully considered to balance cytotoxic effects while reducing the risk associated with cytokine release. High affinity for CD3 has been associated with T cell activation and cytokine release, independently of TAA engagement.

Unlike antagonistic mechanisms of action, which can arise at the target epitope due to steric hinderance, immune engagement antibodies require specific epitopes to present Fc favorably.^132^ Similarly, the proximity of the epitope, and thus the bound antibody, to the target cell surface is a critical determinant of the effector cell and complement activity.^133^

### Agonist antibodies

Agonist antibodies exploit their bivalent nature to facilitate clustering of their targets either within a target cell membrane or in solution.^134^ In this way, they may mimic naturally occurring ligands to induce receptor activation or cross-linking of their targets.

Agonist activity can be studied in a very similar way to antagonist activity but is dependent on the expected consequences of target clustering. This consequence, for example a signaling event such as receptor phosphorylation, could be measured directly or, in the case of co-stimulatory receptors, the ability of the agonist antibody to potentiate the response of another immune cell engaging the antibody can be measured.

The relationship between antibody affinity and agonistic activity has not been as widely investigated as for antagonistic antibodies. A study investigating the affinity/function relationship of agonistic antibodies targeting three different receptors (PD-1, CD40, and 4-1BB), which require clustering in order to elicit their agonistic activity, showed a bell-shaped relationship between affinity and function and that low rather than high affinity, driven by a faster off-rate, resulted in the greatest agonism.^135^ Another study found that the agonistic activity of anti-Fas antibodies was inversely related to affinity and hypothesized that partial dissociation of the antibody is required for receptor activation, a mechanism also driven by a faster off-rate.^136^ Other properties of antibodies beyond affinity have also been observed to influence potency *in vitro*, including hinge flexibility and consequent effects on Fab conformation.^137^

### Antibody-drug conjugates

ADCs are clinically proven therapeutics that deliver a drug payload selectively to target cells. Their primary mechanism of action involves the recognition of target extracellular antigens on specific cell types. Once internalized *via* endocytosis, the drug payload is released. Currently, marketed ADCs are exclusively used in oncology, where they leverage the overexpression of cell surface antigens recognized by the Fab arms, enabling the delivery of cytotoxic payloads with far greater selectivity compared to non-targeted chemotherapeutic approaches.

**Figure 7.**
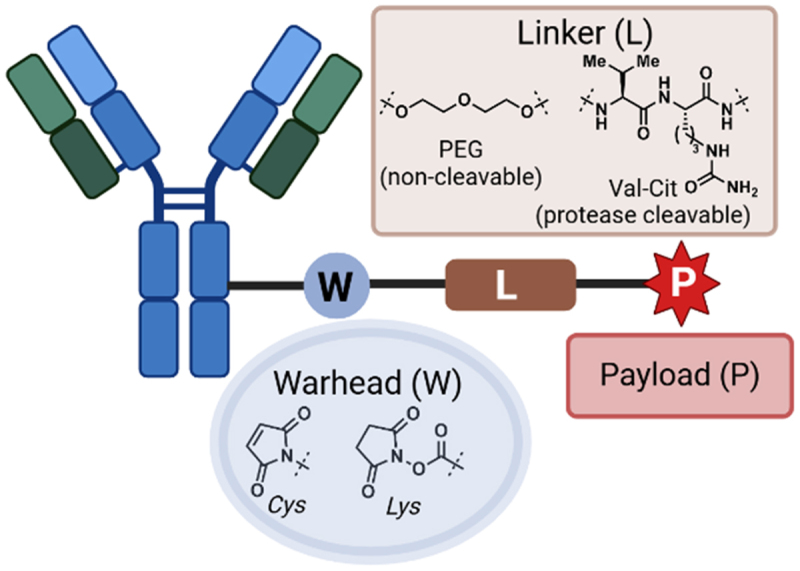
Schematic of an antibody-drug-conjugate and their requisite components.

The general structure of an ADC comprises several structural components (Figure 7). First is the site of attachment to the antibody, featuring an electrophilic warhead that typically occurs at sites of lysine (Lys) or cysteine (Cys) residues.^138,139^ The linker moiety of the warhead-linker-payload (WLP) is inherently tuneable, allowing for customization based on the requirement for solubilizing moieties and the desired drug payload release PK profile.

The structure of ADCs is complex, and their efficacy is influenced by their physicochemical properties and biological features such as cell internalization,^140^ payload release, and mechanism of action of the payload.^141^ Conjugation of the WLP to an antibody, either *via* covalent or non-covalent^142,143^ modification, alters the physicochemical profile of the ADC. Therefore, analyzing the binding profile of the Fab arms for the target antigen is essential for their development as drug candidates and to minimize off-target toxicity.^144^

For example, Zwaagstra *et al*. used a range of biophysical and cell-based assays to conclude that higher affinity anti-Her2 mAbs correlated with higher cytotoxicity in Her2+ cell lines.^141^ However, this higher affinity also resulted in an increased incidence of off-target toxicity, highlighting the need to reconcile cell targeting properties with on/off-target cytotoxicity using an integrated approach using both biophysical and cell-based assays.

Reconciling ADC properties with efficacy can be challenging, especially with Lys conjugation strategies that form a heterogeneous range of drug-to-antibody ratios. Vasic *et al*. recently reported an adaptation of the earlier Format Chain Exchange technology (FORCE)^145^ approach, termed pair-FORCE, to identify optimal ADC candidates.^146^ First, a library of Fab/Fv binder molecules is prepared from a focused pool, and then their sequences are exchanged with Fc fragments conjugated with drug payloads. This enables the functional correlation of an Fc-WLP module with the Fab/Fv fragments. This screening technique will enable fine-tuning of ADC candidates by reconciling their binding properties through a combination of engineering approaches and biophysical analyses.^147,148^ Further innovative uses of biophysical platforms integrated with cell-based assays will open up new opportunities to correlate binding profiles of ADCs with other biological phenomena, such as rates of cellular uptake, cargo release, and receptor recycling.

### Therapeutic Implications for PK/PD

*In vivo*, the pharmacokinetics and pharmacodynamics of an antibody are much more closely entwined compared to a new chemical entity (NCE), i.e., a small-molecule drug, and this is due to the much higher affinity interaction between antibody and target antigen, and the fact that at typical clinical doses the concentration of antibody and target can be more similar than that for NCEs. Overall considerations for antibody design with regard to PK/PD have been reviewed elsewhere,^149^ and we focus specifically on target engagement.

#### Pharmacokinetics

On binding an antibody, the inherent kinetics of a target can become altered. For example, antibody binding can influence the PK of soluble targets by blocking their usual routes of clearance, such as receptor interaction, and the antibody:cytokine complex is too large for renal clearance. This reduced clearance can result in the accumulation of the total target, both free and in the complex with the antibody.^150^

The extent to which this occurs for a given dose of antibody *in vivo*, and the extent to which this accumulation influences the therapeutic aim of free target reduction, is dependent upon the K_D_ of the antibody (the free to total target ratio equals the antibody to K_D_ ratio). This accumulation of the total target can be so large that, after a transient reduction, the free target returns to the baseline. To some extent this issue can be solved by identifying an antibody with a greater affinity, but even with very tight binding, it can be the case that stoichiometry wins – at least as much antibody needs to be bound as the target is produced in the body over the dosing interval.

#### Target-mediated drug disposition

In some cases, the size and function of a soluble target antigen can dictate the elimination rate of its complex with the antibody, rather than the antibody itself, leading to the observation of target-mediated drug disposition (TMDD).^151^ A notable example is antibodies targeting PCSK9, where the overall antibody kinetics is non-linear, with a short elimination half-life.^152^ The PK profile of PCSK9 was modified by engineering CDRs to exhibit lower affinity at an endosomal pH of 6.0, allowing the antibody to disassociate from the target in the endosome and be released from the cell.^153^ There are now several examples of antibodies engineered with a “pH switch” mechanism. This enables the antibody to more efficiently release the target into the endosome before the antibody itself is recycled out of the cell.

TMDD may also occur with membrane-bound targets, where the target antigen either naturally internalizes or is induced to do so when bound by an antibody. The impact on the antibody is determined by both the whole-body expression of the antigen relative to the antibody dose, as well as the internalization rate of the antibody:antigen complex. In simple terms, complex internalization results in a non-linear clearance pathway, where the clearance rate (V_max_) is closely related to the product of the antigen expression and internalization rate, and the half-maximal rate concentration (K_m_) is related to the antibody affinity and internalization rate.^154^ This can reduce the extent of target occupancy on the cell membrane, potentially limiting the effectiveness of antibodies that act as receptor antagonists or elicit immune-mediated mechanisms such as ADCC, yet ensuring selective delivery of ADC payloads to antigen-expressing cells.

#### Antibody distribution

Antibody affinity can influence tissue distribution and the effectiveness of an antibody in reaching its target site. For example, the binding site barrier hypothesis suggests that high-affinity antibodies are less able to penetrate into solid tumors as they become entrapped in outer, antigen-dense sections of the tumor.^155^ Beyond this local effect within a single tumor, the hypothesis extends to the entire body, with high-affinity antibodies exhibiting altered distribution.^156^

Antibody:antigen interactions can be harnessed to improve the tissue distribution of therapeutic antibodies. A notable example is brain shuttling, which enhances central nervous system penetration. In this approach, the antibody is engineered to include a binding domain for a receptor that mediates transcytosis of endogenous ligands, with the transferrin receptor being the most commonly utilized.^157^ Such strategies could significantly improve treatments for conditions such as Alzheimer’s disease.^158^

#### Pharmacodynamics

Pharmacodynamics is the study of the effects of a therapeutic *in vivo*. As highlighted above, for therapeutic antibodies, pharmacokinetics, and pharmacodynamics may be closely intertwined *via* TMDD. The impact of the target on PK can be either deleterious (antagonists and immune agonism) or beneficial. Furthermore, the expression of the target *in vivo*, relative to that used for *in vitro* testing will have a profound impact on the *in vivo* potency of an antibody. While potency is related to the affinity of the antibody for its target, increased physiological target concentration, commonly seen in the disease state, will increase the concentration of antibodies required to achieve a therapeutic effect.^159,160^

#### Hook effect

Overcrowding, or “auto-inhibition”, is a phenomenon associated with multivalent, multi-functional molecules. This can result in the “hook effect” where effect is reduced at higher drug concentrations, as is often observed for proteolysis-targeting chimeras (PROTACs) *in vitro*.^161^ Here, at higher concentrations, binary complexes with either target are formed that block the subsequent formation of the functionally active ternary complex. This is also a consideration for ADCC, where FcγR binding renders antibodies bi-functional, and for T-cell and NK-cell engagers where a hook effect is sometimes observed.^162–165^ The shape of the biphasic concentration-effect (PK/PD) curve is dependent upon affinity and expression of the antigens and therefore lends itself to mathematical model-based optimization.^166^

Bispecific antibodies are monovalent with respect to two soluble targets, and it can be assumed that the binding of either arm is independent. This means that, for a given dose of antibody, the number of binding sites for a target relative to its monospecific parental is halved. Consequently, the effective dose of the bispecific antibody is double the greatest of the two bivalent “parental” antibodies from which it was derived. Despite this, combining two parental antibodies into a single molecule can make the path to regulatory approval simpler, as the safety and efficacy of only a single molecule needs to be evaluated, as opposed to the two parentals from which it was derived.

### Fc engineering

While this review has focussed on the interaction between antibodies and their antigen, it would be remiss not to consider the impact of antibody engineering in the Fc region to enhance or abrogate binding to FcγRs, neonatal Fc receptor (FcRn), and other elements of the immune system.

#### Fc gamma receptors

Antibody affinities for FcγRs are typically low, ensuring that activation only occurs in the presence of high-density immune complexes. Sequence engineering strategies have been well characterized to enhance or abrogate binding to FcγRs, depending on the desired therapeutic effect.^15,16^

In the case of antigens that are expressed at low levels on target cells, enhancement in FcγR engagement by increasing affinity for FcγR has been demonstrated to reduce the antigen expression required to achieve similar activity to antibodies with a wild-type (WT) Fc by up to 10-fold.^167^

It may be desirable to “silence” the Fc of an antibody to avoid effector functions entirely, specifically in cases where the desired mechanism of action is direct neutralization. Neutralization occurs as a result of target engagement alone, and therefore activation of Fc-mediated mechanisms such as cytotoxicity and inflammation are undesirable, leading to potential side effects including cytokine release syndrome.

IgGs contain a conserved asparagine at position 297 (termed N297), which can serve as an anchor for carbohydrate chains. The glycosylation state of this residue has been observed to modulate effector function through subtle changes in the interactions between the antibody Fc domains and other proteins, such as Fc receptors and complements.^168^ The N-linked glycan have been extensively characterized, and the structure can vary significantly depending on the expression system. Complete deglycosylation of the antibody Fc, through mutation of N297 or enzymatic cleavage, has been demonstrated to abrogate FcγR binding and therefore effector function, thus demonstrating its essential role in evoking an immune response.^169^ This glycan is postulated to have a role in maintaining the overall Fc conformation, ensuring amino acid residues are correctly positioned for optimal receptor binding. Some data also suggest that glycan–glycan interactions between the antibody Fc and receptors help to stabilize the complex after non-covalent interactions have occurred between proteins. Afucosylation of the glycan, in contrast, significantly improves the affinity of IgG for FcγRIIIa, and therefore ADCC activity.^170^ Despite these observed favorable characteristics *in vitro*, it is worth considering that modification of the core glycan can affect the developability and *in vivo* stability of an antibody molecule.^171^

#### Neonatal Fc receptor

FcRn plays a crucial role in binding to IgG, enabling endosomal recovery and preventing lysosomal degradation. It achieves this through pH-dependent binding, with a low affinity for IgG Fc at serum pH (pH 7.4), and a much greater affinity at endosomal pH (pH 6.0). This mechanism is key to the prolonged half-life of antibodies.

Enhancing FcRn affinity further, through sequence engineering, has been clinically validated to further extend antibody half-life, enabling both reduced dose and less frequent dosing intervals, which can lower costs and improve patient experience.^172^

The interaction between antibodies and FcRn has also been exploited to develop “sweeping” antibodies.^173^ This strategy involves enhancing the overall affinity for FcRn, removing pH-dependence. Consequently, antibodies are able to remain in complex with FcRn and bind to soluble antigens, delivering them to the lysosome for proteolytic degradation.

Reducing antibody affinity for FcRn is usually not desired, due to half-life implications. However, several therapeutics are under investigation that target FcRn, with a view to depleting native, pathogenic IgG in autoimmune conditions, by way of competing for binding.^174,175^

## Conclusions and outlook

Extensive preclinical assessment of antibody therapeutics, beginning with their target engagement and later their function, is essential to maximize chances of clinical success. Target engagement can be modulated in both a sequence- and structure-based manner and optimized for selectivity and specificity depending on the desired therapeutic outcome.

Various methods have been used to characterize antibody target engagement, with the selection of a method typically being based on the nature of the target antigen, with a view to maximizing physiological relevance and probability of clinical translation. Researchers should be mindful when selecting technologies to assess target engagement, being aware of the limitations of each system and experimental artifacts that may arise and mislead efforts to optimize this. Beyond the assay platform, reagent selection (i.e., antigen) and fitting models should all be carefully selected based on their suitability.

New assay technologies and novel applications of more classical pharmacology are being actively developed to provide multi-parametric datasets and deeper insights into how these molecules engage their targets and other effector cells. Looking forward, the potential of *in silico* tools to predict antibody affinity from sequence alone remains to be seen, making *in vitro* validation critical.

In the realm of small-molecule discovery, it is generally accepted that high affinity results in enhanced function due to increased binding at low concentrations. This early-stage affinity profiling strategy has been extended to large-molecule discovery for similar reasons. However, the multivalent structure of antibodies and their potential for avid-binding interactions, as well as the necessity for multi-protein complexes in certain mechanisms, add nuance to this assumption. Furthermore, the individual association and dissociation constants, from which affinity is derived, are crucial for understanding the assembly and disassembly of immune complexes and antibody function.

Several examples shared in this review highlight the necessity of understanding the target antigen, the disease biology, and the desired mechanism of action. With this information, it becomes easier to understand the desired therapeutic profile of the molecule being developed, including its affinity and molecular format. This understanding also enables the development of mathematical models to describe disease biology and the corresponding target product profile.

Antibody therapeutics are typically administered *via* subcutaneous or intravenous routes. Therefore, it is essential to consider the effects of target engagement on PK/PD to minimize dose and administration frequency. A balance must be achieved between high target engagement and avoiding prolonged residence time that can impact therapeutic efficacy. Recent advances in antibody engineering and computational approaches when implemented in parallel offer the scope to address existing grand challenges in antibody discovery.

## Abbreviations

ADCC: Antibody-dependent cell-mediated cytotoxicity
ADCP: Antibody-dependent cell-mediated phagocytosis
CDC: Complement-dependent cytotoxicity
ADC: Antibody-drug conjugate
AI/ML: Artificial intelligence/Machine Learning
K_D_: Dissociation constant
IC_50_: Half-maximal inhibitory concentration
NGS: Next generation sequencing
IgG: Immunoglobulin G
FcγR: Fc gamma receptor
mAb: Monoclonal antibody
bsAb: Bispecific antibody
CDR: Complementarity-determining region
TCE: T cell engager
Fab: Fragment antigen binding
Fc: Fragment crystallisable
PK: Pharmacokinetics
PD: Pharmacodynamics
scFv: single-chain variable fragment
ITAM: immunoreceptor tyrosine-based activation motif
ITIM: immunoreceptor tyrosine-based inhibitory motif
SPR: Surface Plasmon Resonance
KinExA: Kinetic Exclusion Assay
NCE: New Chemical Entity
